# Parvalbumin: A Major Fish Allergen and a Forensically Relevant Marker

**DOI:** 10.3390/genes14010223

**Published:** 2023-01-14

**Authors:** Subham Mukherjee, Petra Horka, Kamila Zdenkova, Eliska Cermakova

**Affiliations:** 1 Institute for Environmental Studies, Faculty of Science, Charles University, Benatska 2, 128 01 Prague, Czech Republic; 2 Department of Chemistry, Biochemistry and Food Microbiology, Food Research Institute Prague, Radiová 1285/7, 102 31 Prague, Czech Republic; 3 Department of Biochemistry and Microbiology, University of Chemistry and Technology, Prague, Technická 5, 166 28 Prague, Czech Republic

**Keywords:** parvalbumin, fish identification, allergen, gene diversity, forensic marker

## Abstract

Parvalbumins (PVALBs) are low molecular weight calcium-binding proteins. In addition to their role in many biological processes, PVALBs play an important role in regulating Ca2+ switching in muscles with fast-twitch fibres in addition to their role in many biological processes. The PVALB gene family is divided into two gene types, alpha (α) and beta (β), with the β gene further divided into two gene types, beta1 (β1) and beta2 (β2), carrying traces of whole genome duplication. A large variety of commonly consumed fish species contain PVALB proteins which are known to cause fish allergies. More than 95% of all fish-induced food allergies are caused by PVALB proteins. The authentication of fish species has become increasingly important as the seafood industry continues to grow and the growth brings with it many cases of food fraud. Since the PVALB gene plays an important role in the initiation of allergic reactions, it has been used for decades to develop alternate assays for fish identification. A brief review of the significance of the fish PVALB genes is presented in this article, which covers evolutionary diversity, allergic properties, and potential use as a forensic marker.

## 1. Introduction

Parvalbumins (PVALBs) are small-size calcium-binding proteins with molecular weights ranging from 10–12.5 kDa related in structure to calmodulin and troponin C. They are generally expressed in the highest quantity in fast-twitching muscles but are also expressed in other tissues and organs such as the brain and gonads of fishes [1,2]. PVALBs were first discovered in fish and amphibian muscle fibres in 1934 by Deuticke [3] and were later crystallised by Henrotte in 1952 [4] from carp muscle [1]. Despite being called “parvalbumin” by Pechère et al. [5] because of its low molecular weight and high solubility in water, it has no functional similarity to the protein serum albumin. In 1971, Pechère et al. highlighted its binding affinity towards Ca^2+^ [6]. In the following year, the 3D structure of PVALB, the first protein capable of binding calcium, was published [7]. 

Parvalbumins play a critical role in many biological processes. An essential function of PVALBs is to regulate the intracellular Ca^2+^ exchange in fast-twitch muscle fibres. The PVALB proteins are acidic (intracellular isoelectric point, pI: 4.1–5.2) and have a high affinity for Ca^2+^ and can bind two Ca^2+^ ions per molecule [8,9]. PVALB’s also aid in the relaxation process of fast-contracting muscles in vertebrates by carrying Ca^2+^ from troponin C to the sarcoplasmic reticulum via the ATPase pump [10]. Regulating the process is really important because, if Ca^2+^ switching in muscle fibres is left unchecked, it can cause shifts in Ca^2+^ homeostasis, ultimately leading to significant health issues such as Alzheimer’s in humans [11]. Moreover, PVALBSs have been observed to contribute to a variety of swimming forms in fish. Swimming form is a specific pattern of swimming behavior, such as a fast start or a C-bend [8,12]. In fish, the PVALB content in muscle varies from 0 to >1.5 mmol per litre [13]. In addition, the muscle relaxation rate varies longitudinally within a fish due to the variation in PVALB expression along its length [14]. Apart from this, PVALBs have also been detected immunohistochemically in non-muscle tissues, including bone, teeth, skin, brain, seminal vesicles, testes, and ovaries [2,15]. The parvalbumin protein belongs to the calcium-binding protein family of food allergens [16], and it has the ability to survive high temperatures such as many other food allergens [17,18], as well as enzymatic digestion and food processing systems [19]. The IgE reactivity of PVALB is, however, reported to decrease when the tissues are heated to 140 °C, as well as when various seafood processing methods are used [20,21].

The PVALB gene also provides an interesting marker for fish identification. The highly conserved four exons and three introns make it an appropriate tool for the authentication of fish species, as well as serving as a tool for forensic applications in case of fish frauds such as species substitution. This genomic marker provides an alternative to mitochondrial-based markers used for identification, which can be highly useful when comparing closely related fish species [22,23,24,25]. 

Fish muscles usually express 2–5 PVALB isoforms in their white muscle all through development from larval to adult forms [8], whereas a maximum of seven PVALB isoforms were detected in the white muscle of the adult common snook, *Centropomus undecimalis* [26]. Genetic polymorphism of PVALBs was observed in various fish species, including *Cyprinus carpio, Carassius cavatus, Acanthopagrus schlegeli, Tinca tinca*, etc. [2,27]. However, due to the wide-ranging distribution, species-specific expression, and unique electrophoretic mobility, together with exceptional stability, make PVALBs an excellent promising molecular marker for species identification [1]. 

**Figure 1:**
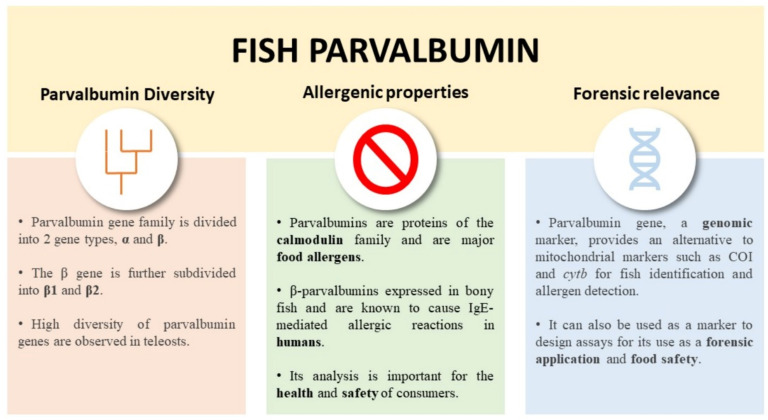
Overview of the importance and applicability of fish parvalbumin.

This article reviews the significance of fish *PVALB* genes. We provide a brief summary about the fish PVALB evolutionary lineage and diversity in teleost fishes. We will also briefly describe the allergenic properties of PV, and its potential as a marker for fish identification, detection of allergens, and forensic application (Figure 1).

## 2. Parvalbumin Gene Diversity

**Figure 2:**
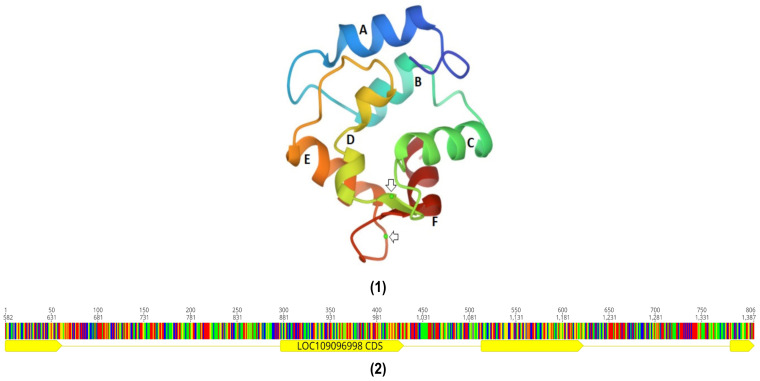
Parvalbumin structure. (1) Carp parvalbumin beta protein ribbon structure. Six helixes (A, B, C, D, E, F) and two Ca2+ ions (Arrow) [35]; (2) Carp parvalbumin gene structure. Four exons (Yellow) and three introns [36].

The *PVALB* gene family is divided into two gene types, alpha (α) and beta (β), with the β gene further divided into two gene types, beta1 (β1) and beta2 (β2). These two phylogenetically distinct gene types of PV, α and β, have different isoelectric points (α, pI > 5.0; β, pI < 4.5). Since *PVALB*s belonging to the α-gene type have fewer amino acid residues than the β-gene type, they have higher isoelectric points than the β-gene type. They also differ in their amino acid sequences [28,29]. While α-genes only express 95-111 amino acid residues, β-genes express 106-113 amino acids [30]. They also have different crystal structures, and physiological roles, as well as magnesium and calcium ion affinities. For example, *PVALB*s of the β-gene type bind more effectively to Ca^2+^ ions than α-lineage *PVALB*s (200% better affinity), but in the case of Mg2+ ions, β-gene type *PVALB*s only have a 16% better affinity [31,32]. In the case of fishes, bony fishes predominantly express β-*PVALB* in muscle tissue while cartilaginous fishes (e.g., rays and sharks) express α-*PVALB*s in muscle tissue, resulting in lower allergic incidence in cartilaginous fishes. Through X-ray diffraction spectroscopy, it has been shown that the PVALB protein can be divided into three domains: AB, CD, and EF. While AB domains play a crucial role in protecting the hydrophobic core of the protein as well as hydrophobic parts of the functional EF hands from solvents, CD and EF domains are involved in the calcium-binding system (Figure 2) [33]. The EF domain is the most well-defined and has been used to characterise the canonical EF-hand Ca^2+^ binding motif [34]. 

Regardless of overall structural similarity to β genes, α-*PVALB* is generally non-allergic in fishes. However, α-*PVALB*s in frog, chicken, and crocodile meat also act as allergens in humans. While sequencing has revealed that chances of cross-reactivity between fish *PVALB*s with its mammalian or avian homologs is low and IgE cross-reactivity is unlikely it is still advisable for sensitized consumers to ascertain caution when consuming these products [37]. On the molecular level β isoforms also differ significantly from α isoforms. For example, alignment of the sequencing data of three *PVALB* gene types of Atlantic salmon (*Salmo salar*) reveals that a higher similarity is observed between the two β-gene types than between the α-gene and either β-gene types. When *PVALB* β1 and β2 were compared amongst each other 71.8 % nucleotide similarity was observed. However, the similarity was significantly reduced to 61.1 % when *PVALB* α and *PVALB* β isoforms were compared [2]. 

**Figure 3:**
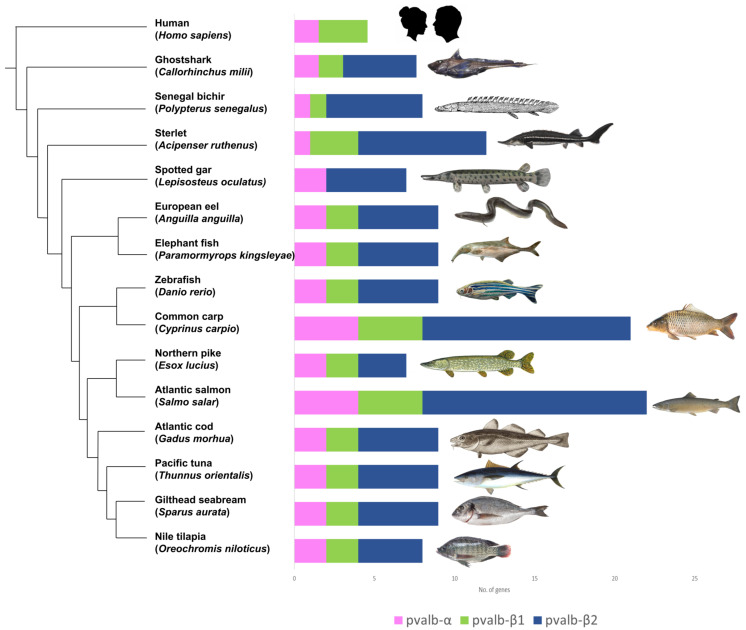
Phylogentic relation of parvalbumin in selected species of the teleost fishes and a non-teleost outgroup. Parvalbumin genes found in the selected fish genomes are shown in groups and coloured by the parvalbumin gene type, i.e., *PVALB*-α in pink, *PVALB*-β1 in green, and *PVALB*-β2 in blue The phylogenetic relation is according to the work of Mukherjee et al. [2].

The parvalbumin gene family, such as that of the *HOX* gene family, is a conserved gene and carries traces of whole genome duplication [38]. The origin of *PVALB*-α, *PVALB*-β1and *PVALB*-β2 gene types have been attributed to vertebrate ancestors [39]. The diversity of *PVALB* genes is a possible result of the occurrence of vertebrate-specific whole genome duplication. In a recent study by Mukherjee et al. [2], a high diversity of *PVALB* genes was observed in teleost fishes. Apart from ancestral vertebrate gene duplication, several teleost lineages such as the Salmoniformes, Cyprinidae, and Sturgeons underwent additional lineage-specific duplication events giving rise to even more clusters of *PVALB* genes with higher diversity. The study reported a variable number of *PVALB* gene copies within the teleosts, from seven copies in *Esox Lucius* (Pike) to 22 copies in *S. salar* (Atlantic salmon) (Figure 3).

The diversity of the three *PVALB* genes was also observed among non-bony fish vertebrates such as *Callorhinchus milii* (ghost shark), *Gallus gallus* (chicken), *Homo sapiens* (human), *Rattus norvegicus* (mouse), and *Xenopus laevis* (frog). Amongst the three gene types, the *PVALB* β2 gene was the most diverse in teleost fishes, with salmon possessing up to 14 copies of the ancestral *PVALB* β2. *Homo sapiens* (humans) possess one *PVALB* α, one *PVALEF*, two oncomodulin {OCM (OCM, OCM2)}, and three calmodulin (CALM) genes. Oncomodulin genes OCM and OCM2 are both similar to the fish *PVALB* β1 gene. Belonging to the EF-hand protein family, oncomodulin genes express proteins to increase the calcium-ion binding affinity. These proteins are also found in early embryonic cells in the placenta and can also be found in tumors [40].

While this gene (*PVALB* β2) is most diverse in teleost fishes, it is absent in mammals such as humans and mice [2]. Due to the presence of all three isoforms in a single species, as observed in teleost’s fishes, a layered complexity is added for diagnosing, detecting, and effectively managing allergic *PVALB* [41]. So, albeit having α, β1, and β2, the gene types are also present in multiple copies. Phylogenetic analysis along with structural and biochemical investigations have assigned α and β *PVALBs* as separate clusters [2]. While the α subtype is present in humans and other vertebrates such as the house mouse (*Mus musculus*), subtype β2 is absent in them. The allergenicity of these two gene types may be due to the distantly separated clusters of *PVALB*s.

## 3. Parvalbumin—A Major Fish Allergen

Food allergy is an immunologically based adverse reaction to food or food additives. Allergies caused by foods are deemed a significant hazard to public health, particularly for those sensitive to an allergic reaction. Peanuts, soy, milk, shellfish, fish, and tree nuts are some common foods that induce allergic reactions [42]. These food sources contain high levels of allergens, which remain stable when processed and digested in the body [43]. While some allergens degrade during the process of digestion, their fragments are still identified by IgE antibodies that instigate an allergic reaction [44].

Nowadays, fish allergy is one of the most frequently occurring food allergies among children and adults [45]. Fish is an integral part of the human diet and nutrition since it is rich in essential lipid-soluble vitamins, polyunsaturated fatty acids (such as docosahexaenoic acid and eicosapentaenoic acid), and essential amino acids. Even though fish intake in landlocked countries continues at a reasonably stable state, the overall demand for fish and fish products continues to increase worldwide [45,46]. 

PVALBs are responsible for more than 95% of fish-induced food allergies [28]. Studies provide evidence that the PVALB protein is a fish allergen for a wide range of commonly consumed species, including salmon, carp, mackerel, tuna, and pilchard [47,48]. Due to the high cross-reactivity of β-PVALB from different species, more than 90% of people sensitive to fish usually have allergic reactions to several species of fish. Researchers found a 50% possibility of reacting to PVALB from more than one fish species [49,50,51]. The allergen cross-reactivity can be evaluated by comparing amino acid sequences of isoallergen. While certain fishes such as *S. salar* (Atlantic salmon) and *Rastrelliger kanagurta* (Indian mackerel) possess only one PVALB isoallergen, others such as *Gadus morhua* (Atlantic cod), *Lates calcarifer* (Baramundi), and *Clupea harengus* (Atlantic herring) contain more than one (Table 1). Fish species that are closely related also exhibit marked clinical cross-reactivity. A significant number of food-allergic individuals may suffer severe health problems due to unintentionally consuming products containing undeclared seafood [52]. Cross-reactive PVALB epitopes are located in highly conserved protein regions, especially at the ion-binding sites [53]. Oral allergy syndrome and rhinitis are general clinical manifestations, as well as diarrhea, abdominal pain, angioedema, urticaria, asthma, and, in severe cases, life-threatening anaphylactic reactions. 

For allergy sufferers, the only effective way to prevent an adverse reaction in the event of exposure to the allergenic food is to avoid seafood altogether or, in the case of accidental exposure, to use therapeutic treatment (e.g., antihistamines, corticosteroids, epinephrine) [54]. However, it has been shown that even patients with extreme fish sensitivities can consume certain kinds of fish, such as tuna, without an untoward event [55,56]. In addition, molecule-specific epitope regions are known, such as PVALBs from salmonid fish, explaining a limited cross-reactivity with these species. Nonetheless, there are substantial differences in PVALB content between fish species, and these variations correlate with differences in the allergenicity of fish PVALBs [28,57]. For example, Kuehn et al. [28] demonstrated that the PVALB level in fish muscles is up to 100 times higher for carp than mackerel or tuna. They found an average of <0.05 mg/g PVALB in tuna; 30.7 mg/g in mackerel; 12.5 mg/g in salmon, trout, and cod; and >2.5 mg/g in carp, herring, and redfish. The amount of PVALB depends not only on the fish species but also on the method of preparation. PVALB allergenicity decreases due to fish processing through cooking, baking, and smoking; due to this variability, people with a clinically relevant sensitization to PVALB may still eat processed fish with a lower concentration of PVALB without a reaction. In the case of fish allergen PVALB, isoforms and the extent of thermal processing influence antibody reactivity. Saptarshi et al. [18] validated this by comparing PVALBs in a wide-range of fishes (raw and heated fish extracts) from the Asia-Pacific region through immunoblotting experiments. They found variations in the thermal stability of PVALB within the tested fish genera, and their results demonstrate that heat processing the antigen significantly affects the reactivity of antibodies to PVALBs. Human antibodies reacted less strongly to multimeric bony fish PVALBs after heating, whereas antibodies lost reactivity completely in cartilaginous fish. 

Apart from clinical advances, it has become imperative to improve consumer protection through an accurate food labelling system preventing potentially life-threatening risks for sensitized/allergic individuals [58]. According to recent European Union (EU) regulations, food manufacturers are required to declare the presence of 14 food groups classified as potentially allergenic, namely fish, crustaceans, molluscs, celery, mustard, sesame seeds, gluten, tree nuts, peanuts, milk, eggs, soybeans, lupins and sulphites, and highlighting them from the list of other ingredients [59].

As of 2022, there are more than 290 entries in the Allergome database (www.allergome.org) for fish PV and its isoforms and allergens [60]. Out of these 290, 27 isoforms from 17 fish species are registered and documented with the World health organization (WHO) and the International Union of Immunological Societies (IUIS) (Table 1).

**Table 1:** Fish and non-fish parvalbumin allergens registered with the WHO (www.allergen.org (accessed on 15 November 2022)).

Name of Allergen	Organism		Biochemical Name	Genbank Nucleotide Accession no.	Reference
	Scientific Name	Common Name			
Fish species					
Clu h 1	*Clupea harengus*	Atlantic herring	β-parvalbumin	FM178220	[61]
				FM178221	
				FM178222	
Cten i 1	*Ctenopharyngodon idella*	Grass carp	β-parvalbumin	MK140606	[62]
Cyp c 1	*Cyprinus carpio*	Common carp	β-parvalbumin	AJ292211	[63]
				AJ292212	
Gad c 1	*Gadus callarias*	Baltic cod	β-parvalbumin		[64]
Gad m 1	*Gadus morhua*	Atlantic cod	β-parvalbumin	AY035584	[65]
				AM497927	
				AY035585	
				AM497928	
lat c 1	*Lates calcarifer*	Baramundi	β-parvalbumin	AY688372	[66]
				KF021278	
				AY626068	
				KF021279	
				AY688373	
Lep w 1	*Lepidorhombus whiffiagonis*	Megrim, whiff, turbot fish	β-parvalbumin	AM904681	[19]
Onc m 1	*Oncorhynchus mykiss*	Rainbow trout	β-parvalbumin	not specified	[67]
Pan h 1	*Pangasianodon hypophthalmus*	Striped catfish	β-parvalbumin	XM_026916202	[68]
				XM_026947968	
Ras k 1	*Rastrelliger kanagurta*	Indian mackerel	parvalbumin	KX527884	[69]
Sal s 1	*Salmo salar*	Atlantic salmon	β-parvalbumin 1	X97824	[70]
Sar sa 1	*Sardinops sagax*	Pacific pilchard	β-parvalbumin	FM177701	[47]
Sco s 1	*Scomber scombrus*	Atlantic mackerel	parvalbumin	FM994926	[48]
Seb m 1	*Sebastes marinus*	Ocean perch, redfish	β-parvalbumin	FM178218	[71]
				FM178219	
Sole s 1	*Solea solea*	Sole	parvalbumin		[72]
Thu a 1	*Thunnus albacares*	Yellow fin	β-parvalbumin	FM178217	[73]
Xip g 1	*Xiphias gladius*	Swordfish	β-parvalbumin	FM202668	[19]
Non-fish Species					
Cro p 1	*Crocodylus porosus*	Australian saltwater crocodile	β-parvalbumin	XM_019542160	[74]
Cro p 2			α-parvalbumin	XM_019544844	
Gal d 8	*Gallus domesticus*	Chicken	α-parvalbumin	FM994924	[75]
					[76]
Ran e 1	*Rana esculenta (Pelophylax esculentus)*	Edible frog	α-parvalbumin	AJ315959	[77]
Ran e 2	*Rana esculenta (Pelophylax esculentus)*	Edible frog	β-parvalbumin	AJ414730	

## 4. Forensic Application of Parvalbumin

The seafood industry is ever-increasing and as the industry expands, the issue of authentication of fish species becomes more imperative. With the increase in fish consumption and an uncertain supply and demand chain, cases of the surreptitious substitution of one species with another (fish fraud) are on the rise. While fish fraud not only amounts to economic deception, it can also have a detrimental effect on consumers’ health due to species-specific antigenicities discussed above and environmental management programs for endangered species. Regulatory authorities such as US FDA, FAO, and EU have established laws for labelling fish products to prevent product substitutes, however, these regulations can be difficult to enforce when morphological fish identification is not possible. Hence, apart from the enforcement of labelling regulations, research into various analytical methods for fish identification is carried out to overcome challenging situations such as precooked and frozen seafood. 

Fishes are particularly unidentifiable through their external features in the case of landlocked countries since most fish is imported in the form of compact frozen blocks of meat or fillets [78]. Taxonomical identification is an ideal method for identifying fish species to prevent adulteration of fish, however, it is compromised when distinguishing features are removed (for example, head, fins, scales, and skin in fish) or if the specimen has been cooked. It may also be difficult to ascertain the geographic origin and also to visually identify fish due to the phenotypic resemblances of some fish species [79]. In many instances, globally, fraudsters take advantage of this fact and intentionally mislabel fish products by substituting a species of high worth with a low-priced alternative. Experts anticipate an increase in cases of food fraud due to COVID-19 regulations since there have been reduced private sector food inspections and audits and limitations in supply and demand [80,81]. Therefore, it is essential that standardized, accurate, and simple fish identification methods should be developed that have global use. 

A number of assays and methodologies have been used to tackle the problem of fish identification in the past few decades. Since the PVALB gene plays an important role in inducing allergenic reactions, it has been used for decades to design assays to be used as an alternate approach for fish authentication. The assays can be broadly divided into protein-based assays that include electrophoretic [22,82], chromatographic [83] or immunological [84] methods, and DNA-based assays (PCR with species-specific or universal primers, DNA microarray) [85,86,87,88], and biosensor based assays [89,90].

### 4.1. Protein-Based Assays

Parvalbumin proteins can be regarded as a suitable biomarker for fish species authentication and have been detected and quantified using a variety of protein-based assays [91]. PVALB proteins are expressed in high concentrations in fish muscle and also have high interspecies variability. The interspecies variability of PVALB sequences is fundamental for the discrimination of different fish species as assessed by earlier proteomic analyses performed on species belonging to the Merlucciidae family (hake’s) [92]. PVALB solubility in aqueous buffers makes the extraction protocol both easy and extremely quick. The structural stability of PVALBs even under harsh conditions such as heat is paramount to utilising these biomarkers also for the authentication of fish species sold as thermally processed products [93,94].

Earlier, isoelectric focusing (IEF) was one of the most widely used methods for fish identification [95]. Urea IEF and IEF in immobilized pH gradients (IPG) have great practical significance in the analysis of both fresh and boiled samples. However, in urea IEF, standard marker proteins cannot be used due to modification of their conformation, which impacts their isoelectric points. [95,96]. In place of standard marker proteins, known fish PVALBs can serve as marker proteins in IEF gels for unknown PVALBs due to their thermostable properties and may also be employed in database generation for the differentiation and identification of diverse fish or other specimens [8]. Dobrovolov et al. [97] demonstrated the ability to differentiate various species of sturgeons and their origin using the IEF of sarcoplasmic protein (general muscle protein such as lactate dehydrogenase, malate dehydrogenase, or malic enzyme). Using a similar principle, PVALB was detected in three sturgeon species, *Acipenser baeri*, *A. gueldenstaedtii*, *A. ruthenus* [98]. Authentication of closely related scombrid, catfish, and tilapia species by isoelectric focusing of PVALB also provided a rapid screening method for identification [22]. IEF was also used to study mislabeling in cases of various Alaskan flatfishes in German meat markets [99].

In addition to IEF, tandem mass spectrometry has been used to define the structure of protein isoforms that are essential to understanding cross-reactivity among various allergenic proteins [100]. More recently, exploiting the improved performance of new instruments such as Fourier-transform ion-cyclotron resonance (FTICR) mass spectrometers and linear ion trap (LIT) mass spectrometers, innovative strategies for the extensive characterization of PVs have been proposed. These studies led to the de novo sequencing of 25 isoforms from all commercial species of the Merlucciidae family and the rapid and direct detection of the presence of fish allergens in all of the investigated food products [94,101]. Detection of allergens with high levels of sensitivity was also achieved by employing an optimized protein chip [102]. These assays were useful in detecting fish allergens thus increasing consumer safety.

MALDI-MS can be used in the quality control processes including the main issues of fish authentication and fraud detection. Apart from MALDI-MS, the application of MALDI-TOF in the mass spectra of sarcoplasmic proteins allows the authentication of fish species. Targeted proteomics has been applied to assess fish authenticity and detect allergens. Proteomics offers tools potentially suitable as routine tests in food authentication [103]. PVALBs exhibit a high ionization efficiency in MALDI-TOF MS analysis so that, regardless of the complexity of the analyzed sarcoplasmic extracts, the obtained mass spectra predominantly show signals originating from these proteins [100,104]. Proteomic studies integrating two-dimensional electrophoresis (2-DE) with MALDI-TOF MS peptide mass mapping for protein identification allowed the characterization of the 2-DE PVALB-specific pattern and the definition of a set of specific tryptic peptides suitable for the identification of nine hake species [105,106].

Studies have shown that immunoassays, including indirect ELISA and Western blotting, are the primary tools for detecting fish allergen changes following processing [107]. Despite being primary tools, immunoassays might be inaccurate due to cross-reactivity with nontarget allergenic proteins, and they might not be specific enough to detect allergens [108]. MALDI-MS also have certain disadvantages, such as in some cases, the inability of the system to differentiate between two related species may be due to the inherent similarity of the organisms themselves. Another reason that similar species may be incorrectly identified is a lack of sufficient spectra in the database. If this occurs, it is possible to obtain an incorrect species-level identification or no identification at all [109]. Hence, to overcome the limitations possessed by protein-based methods, DNA-based approaches for fish identification are intrinsically independent of biomolecular interactions and are thus slowly gaining more interest than protein-based methods [110].

### 4.2. Using DNA-Based Assays for Fish Identification 

The genetic identification of species is based on DNA polymorphisms or genetic variations caused by naturally occurring mutations in the DNA [111]. The *PVALB* gene is a conserved gene with highly conserved exons in the protein-coding part of the *PVALB* gene, separated by three introns that are unique among various fish species [88]. *PVALB* can be used as an interesting universal marker for fish identification and allergen detection, as Sun et al. [25] and Rehbein [88] demonstrated. Both universal and species-specific assays have been designed in recent times for better detection of *PVALB* genes (Table 2). 

DNA-based methodologies follow an indirect approach to detecting allergens because they identify the DNA sequences from the allergenic food components rather than detecting the allergenic protein itself [112]. DNA is overall more stable than proteins, particularly when subjected to thermal treatments. Even though DNA may fragment at high temperatures, it is still detectable [25]. DNA-based methodologies thus have a significant advantage over other methods and as a result, DNA-based methods are especially useful to analyze highly processed foodstuffs, emphasizing their important role in the management of allergens in the food industry [113,114]. 

The most frequent markers used for fish identification are found in the mitochondrial genome, such as cytochrome oxidase 1 (COI) and Cytochrome b (Cyt b). Due to the fact that mitochondrial genes are a part of a large number of copies in fish tissues, and their mutation rate is much higher than that of nuclear genes, mitochondrial gene loci are usually relied upon for species identification in fish, primarily as a result of their characteristics such as being part of a haploid genome with a high copy number [88,115]. Due to the fact that mitochondrial genes are small and mitochondria are numerous and relatively easy to isolate, mitochondrial genes are widely used for species identification. Additionally, the number of mitochondrial DNA (mtDNA) gene sequences available in public databases has rapidly increased, making it relatively easy to compare a test sequence with previously identified samples. 

However, in the last few years, markers residing in the nuclear genome have also started to play an essential role in constructing such species-identification assays. Although mitochondrial DNA has quite a few advantages when compared to nuclear DNA, some disadvantages must be considered [116]. Although mtDNA markers can separate species, they can sometimes be uninformative while separating closely related species, such as in the case of *Thunnus* species [117] and in cases of mixed products. The mixed product can be manufactured by combing premium quality tuna with tuna of lower quality and price. The sequencing of mtDNA also has the significant disadvantage of potentially introducing nuclear mitochondrial pseudogenes (numts), which are mitochondrial-derived non-functional nuclear sequences [118,119]. In contrast, amplifying nuclear sequences (markers) can overcome these problems while providing fairly high levels of uniqueness even in closely related fish species [23,88,120].

The identification of certain species has also been proposed using novel nuclear regions, such as the flatfish genome [121]. DNA can be amplified even in highly processed foods since nuclear DNA (nDNA) barcodes tend to be shorter than mtDNA barcodes. Next-generation sequencing (NGS) can be conducted on DNAs extracted from these foods as they are easy to read. As a result, species can be identified even in samples containing several species [121].

**Table 2:** Universal and species-specific primers that have been used for detection of *PVALB* genes.

Organism		Primer Name	Primer Sequence (5′-3′)	Amplification Length (BP)	Amplification Region	Reference
Scientific Name	Common Name					
Universal		IFF232	GACAAGAGCGGCTTCATTGAGG	268	β-parvalbumin	[122]
		IFF233	TCAACTCCAATCTTGCCATCACCAT		Exon 3 to Exon 4	
Universal		IFF 233a	TCAATACCGATCTTGCCATCACCGT	NA		[123]
		IFF 233b	TCAACTCCGATCATGCCATCACCAT			
Universal		SUN-F	CAGGACAAGAGTGGCTTCAT	57	β2-parvalbumin	[25]
		SUN-R	GAAGTTCTGCAGGAACAGCTT		Exon2 to Exon 3	
		probe	AGGAGGAYGAGCT			
*C. harengus*	Atlantic herring Pacific herring	CluHaPaF	CCGCTGATGATGTGAAGAAG	189	β2-parvalbumin	[124]
*Clupea pallasii*		CluHaPaR	GCAGGAACAGCCTGAGAGAG		Exon 2 to Exon 3	
*Cyprinus carpio*	Carp	MA-f	ACAAGCTTATGGCTTTCGCCGGAATTCTGA		β-parvalbumin	[125]
		MA-r	ATCGGATCCTATGCCTTGATCATGGC			
*G. morhua*	Atlantic cod	rGad m 1.01	ATGGCATTCGCTGGAATTCTCG	599		
		rGad m 1.02	ATGGCTTTCGCCGGAATTCTG	797		
*Lophius piscatorius Lophius budegassa*	White anglerfish	DAS-F	ACAACTTTCCCCGAGAAGC	196	β2-parvalbumin	[126]
	Black-bellied anglerfish	DAS-R	ACAACATCACAGTTTAAGTTTTGC		Exon 2 to Exon 3	
*Oncorhynchus mykiss*	Rainbow trout	601F9 forward	AGACAGAGACACAGGTTGGCTTACTATTCT	75	β-parvalbumin	[24]
		601G0 reverse	TTTACGACATAGGGAGCAGCTTACTATTCT		Intron 2	
*Paralichthys olivaceus*	Japanese flounder	sunF	GATGACACCATATGTCTCTGGCATCTAAGCTGTCTG	327	β-parvalbumin	[127]
		sunR	GTGTCCTCGAGTTACTGTTTCACCATCGCCGC			
*S. salar*	Atlantic salmon	601F5 forward	AGACAGAGACACAGGTTGGCTTACTATTCT	126	β-parvalbumin	[24]
		601F6 reverse	TTTACGACATAGGGAGCAGCTTACTATTCT		Intron 2	
*S. salar*	Atlantic salmon	Sense PV	AGYGGCTTYATHGARGARGAYGARYT	430	β2-parvalbumin	[70]
		Antisense PV1	YTGYTTNACNAANACNGCRAAYTC		Exon 2 to Exon 4	
		Antisense PV2	GAATTCRTCRACHCCDATYTTHCC			
*S. salar*	Atlantic salmon	IFF 156	ATGGCCTGTGCCCATCTGTGC	300	β1-parvalbumin	[122]
		IFF 157	GGACTTCGAGGCAAAGCCAAT		Exon 1 to Exon 2	
*S. salar*	Atlantic salmon	Psal1	CTGTGCCCATCTGTGCAAGG	650	β1-parvalbumin	[128]
*Oncorhynchus mykiss*	Rainbow trout	Psal2	CCAATCATGCCATCACCATCG		Exon1 to Exon 3	
*Salmo trutta*	Brown trout	Psal3	TACCGATGCAGAGACAAAGG	931	β1-parvalbumin	
*Salvelinus alpinus*	Arctic char	Psal4	GTCTTGGGCAATATTGTTCC		3′ end of Exon 3 to Exon 4	
*Scomber japonicus*	Mackerel	SJ9	CCCTACAAAGCAAAAACATC	1500	β-parvalbumin	[129]
		SJ487	GCATAGGAGGAAAGGICTCT			
		SJ106	GTAGITTCGACCACAAAAAGTT	190	β-parvalbumin	
		SJG441r	ACTGCTGTATAGGTGATAGG		Exon 2 to Intron 2	
		SJG107f	AGCTATTCTGTATCGCTTCG	284	β-parvalbumin	
		SGG297r	GGTGTGAGTCTTACTTCAGC		Intron 1 to Intron 2	
*S. scombrus* *Trachurus trachurus*	Atlantic mackerel Atlantic horse mackerel	Pval1Fw	CTGAAGCTGTTCCTGCAGAACTT	87	β-parvalbumin	[130]
		Pval1Rev	GCTGTCACCGGCCTTGAG			
		Pval1Probe	[6FAM]TCCGACGCCGAGACCAAGGC[TAM]		Intron 2 to Exon 3	
*Spondyliosoma cantharus*	Black seabream	1189B6	TGAGCTGAAGTAAGACACTCAGGAA	78	β-parvalbumin	[23]
		1189B7	TCTAAAATGTTGTCTTGGTGCCTTAG			
		1273H9(probe)	TGCACACTTGAGCAAGCAATGGCC		Intron 2	
*Spondyliosoma cantharus*	Black seabream	601F7 forward	AGACAGAGACACAGGTTGGCTTACTATTCT	79	β-parvalbumin	[24]
		601F8 reverse	TTTACGACATAGGGAGCAGCTTACTATTCT		Intron 2	
*Thunnus albacares*	Yellowfin tuna	ALB4F	AGGATTGGATTTTCTGTCTTAGCTT	227	β-parvalbumin	[22]
		ALB4R	TCAGTTTGTGTCAATTGGTCTGTAG		Intron 2	
		PARVT1F	GGGGTTGGAGATGAATGGCA	785	β-parvalbumin	
		PARVT1R	GAGTCACCGGCCATGAGAAA		Intron 1 to Exon 3	
		PARVT2F	ACAGCTGCCGACTCTTTCAA	670	Parvalbumin β	
		PARVT2R	CGGCCATGAGAAATGCCTTG		Intron 1/Exon 2 to Exon 3	

NA = Not available in the reference.

Previous reports showed that the PVALB protein of bony fishes could be encoded by different paralogs genes that contain the same number of exons and introns but whose introns differ in size and nucleotide sequence, such as the Parv β1 polymorphic site in salmonoids, which was demonstrated by Muñoz-Colmenero et al. [128]. Each orthologous exon contains an identical number of nucleotides in all the paralogs, for example, the exon sequence of *PVALB* β1 of carp (*Cyprinus carpio*) and rainbow trout (*Oncorhynchus mykiss*) is 330 bp long [131,132,133]. Rencova et al. [124] demonstrated a fast, simple, specific, and sensitive PCR assay for the detection of *PVALB* in two closely related herring species (*C. harengus* and *C. pallasii*). Additionally, *PVALB* species-specific primers were used to authenticate closely related species of scombrid, catfish, and tilapia [22]. These results validate the use of DNA-based assays for cheap, routine screening methods for PVALB allergens in fish and food products.

Conserved *PVALB* exon sequences can be used to design universal PCR primers that amplify a species-specific intron, as well as regions of the exons flanking the intron, from even very distantly related fish species, such that fish species identification could be achieved by using probes that target a species-specific intron region [134]. EPIC (Exon primed intron crossing) PCR makes use of this property demonstrating the suitability of nuclear intron sequences as molecular markers for PCR-based species determination and subsequent real-time PCR-based quantification of the extent of each species in a complex mixed sample [25,88]. The use of the *PVALB* gene for fish identification and their quantification in commercial products is well documented in the literature. Results obtained confirmed the possibility of using the *PVALB* gene for forensic application in the fish trade and food industry.

### 4.3. Methods of Parvalbumin Allergen Quantification

Apart from the traditional assays based on protein and DNA detection, biosensor detection of PVALB for fish authentication is a developing field. Biosensors are integrated receptor-transducer devices that convert biological recognition events into measurable chemical, and physical signals proportional to the target concentration. Depending on the target allergen, the receptor might be an antibody raised against it, a single-strand DNA molecule that hybridizes with the allergen-specific DNA fragment, or an aptamer that recognizesthe allergen directly. To detect PVALB a fluorescence sensor was developed by Jiang et al. [89] exhibiting the possibility of quantification of fish PVALB. They also demonstrated its utility for food allergen and detection. Similarly, kinetic analysis by a surface plasmon resonance biosensor was performed to understand the parvalbumin antigen-antibody interaction, providing a fast and powerful tool for allergen detection and quantification [135]. Recently a gold nanoparticle aptasensor was developed for PVALB detection [136]. While gold-based nanoparticles offer an excellent platform for developing rapid, low-cost, portable biosensors for food safety detection, they also have certain drawbacks. For example, colour changes of gold particles can be difficult to interpret in cases of low concentration. Further stability issues of the sensor may change over time giving inaccurate results [137]. While biosensors provide a good alternative in the field of food safety and allergen detection, it is necessary to address the drawbacks before moving forward. 

## 5. Future Outlook

The *PVALB* gene, usually present in fish’s muscle tissue, is a major fish allergen affecting humans. The presence of three gene types (*PVALB* α, *PVALB* β1, and *PVALB* β2) makes detecting and managing these allergens difficult. A high level of cross-reactivity between species is observed without proper knowledge of the cause of the cross-reactivity. Though significant innovations and efforts have been made in recent times to study and control food allergies, further work is still required. While the scientific community has managed to identify new isoforms successfully, some work is still needed to develop standardised methodologies and assessment tools to detect and quantify PVALBs easily, quickly, and efficiently. Developing reliable, rapid, on-site allergen-detection methods showing high accuracy and sensitivity are future research needs for seafood products that will benefit the food industry and make food products healthier for consumers. However, avoiding allergenic seafood intake is still the only standard way for clinical protection of seafood-allergic patients. Consequently, it is imperative to conduct in-depth research into the therapeutic hypoallergenic treatment of PVALB in order to decrease the risk of seafood allergy and provide a safe environment for global consumers. Studying trends in the incidence of fish allergies is also critical to understanding the burden of allergic disease. Unfortunately, no robust studies have been conducted to assess food/fish allergy prevalence trends over time. This lapse could be the result of the data gap that exists between recorded statistics and real allergy diagnostics based on DBPCFC (Double Blind Placebo Controlled Food Challenge). 

With the increase in cases of fish substitutions and fish fraud, reliable methods are needed to detect and identify fish products. The *PVALB* gene has high variability which makes it a reliable marker for fish identification. *PVALB* assays could be used as a tool to control species mislabeling of samples containing closely related fish species. At the same time, efforts have been made to develop authentication tools for commercial purposes by cataloguing SNPs. There is a need to find out and catalogue more SNPs of species of commercial interest. Since most studies are focused on the northern hemisphere, data available from the southern hemisphere is still sparse. Filling this data gap is essential in cataloguing SNPs enabling the development of more reliable and efficient tools that can be applied around the globe. 

## Data Availability

Not applicable.
